# Dietary diversity and associated factors among children aged 6 to 23 months in Chelia District, Ethiopia

**DOI:** 10.1186/s12887-021-03040-0

**Published:** 2021-12-11

**Authors:** Shambel Keno, Haile Bikila, Tesfaye Shibiru, Werku Etafa

**Affiliations:** 1 West Shoa Zone Health Bureau, Oromia Region, Ethiopia; 2 Department of Public Health, Institute of Health Sciences, Wollega University, Nekemte, Ethiopia; 3 School of Medicine, Department of Pediatrics and Child Health, Institute of Health Sciences, Wollega University, Nekemte, Ethiopia; 4 Department of Pediatrics and Neonatal Nursing, Institute of Health Sciences, Wollega University, Nekemte, Ethiopia

## Abstract

**Background:**

Although minimum dietary diversity (MDD) is one of the core indicators of a high-quality diet for infants and young children, meeting this dietary diversity standard remains a challenge in Ethiopia. Therefore, adequate information on the status and factors affecting minimum dietary diversity is essential to identify potential strategic interventions. This study to study is to assess DD and associated factors among children aged 6 to 23 months in Chelia District, Ethiopia.

**Methods:**

A community-based cross-sectional study was conducted in the seven kebeles of Chelia District from 12th April to April 30th, 2020. Kebele is the smallest administrative unit in Ethiopia. Multistage sampling was used to select 631 participants who had a child aged 6 to 23 months. Data was entered into the Epi data version 3.1 and analyzed using Statistical Package for Social Science (SPSS) version 24. A binary logistic regression was fitted to identify significant factors associated with met MDD at 95% CI and a *p*-value < 0.05.

**Results:**

Less than one-quarter (17.32%) of infants and young children aged 6 to 23 months had met MDD. The dominant group of foods consumed was composed of grains, roots, and tubers. Children aged18-23 months (AOR = 3.26, 1.36-7.79), mothers aged 35-44 years (AOR = 3.25, 1.38-7.45), housewives as household heads (AOR = 3.41, 1.56-2.37), children of smaller family size (AOR = 3.89, 1.18-12.78), and caregivers who studied grade 9-12 (AOR = 9.98, 5.66-17.10), who received information about food diversity during ANC (AOR = 1.48, 2.21-11.23) and PNC (AOR = 3.94, 2.04-7.63) visit, who travels less than one hour to reach the market (AOR = 2.94, 1.24-6.91) and who had high family income (AOR = 4.12, 1.90-8.19) were significantly associated with meeting MDD.

**Conclusion:**

Dietary diversity among children aged 6 to 23 months in Chelia District is low. It is proposed that caregivers on MDD be educated/trained and encouraged to share information during health service in order to increase the diverse diet and achieve a better dietary diversity score for infants and younger children.

**Supplementary Information:**

The online version contains supplementary material available at 10.1186/s12887-021-03040-0.

## Introduction

The nutritional status of children under two years of age is directly affected by their feeding practices and, ultimately, impacted child survival. The World Health Organization (WHO) defined minimum dietary diversity (MDD) as the consumption of four or more food groups from the seven food groups for higher dietary quality and to meet daily energy and nutrient requirements of the seven recommended food groups for children aged 6–23 months. These seven food groups were: grains, roots, and tubers; legumes and nuts; dairy products (milk, yogurt, cheese); flesh foods (meat, fish, poultry, and liver/organ meats); eggs; vitamin-A rich fruits and vegetables; other fruits and vegetables [1]. This cut-off point was associated with the better quality of diets for both children aged 6 to 23 months who are breastfeeding and not [2].

Implementing the core indicators of Infant and Young Child Feeding (IYCF) practices such as early initiation of breast milk with one hour of birth, implementing exclusive breastfeeding for under six months of life, initiating complementary feeding at six months of life, continuing breastfeeding until one year of age, and consumption of iron-rich or iron-fortified foods contributes a significant reduction in child mortality and morbidity [3]. The Mini Ethiopian Demographic and Health Survey (EMDHS) (2019) showed that 59% of infants under six months are exclusively breastfeeding while 36% started foods. The 2016 EDHS reported that 73% of children began breastfeeding within 1 h of birth, 92% within the first birthday, and the practice of prelacteal feeding 8% in 2016. Consumption of foods rich in vitamin A or iron remains low among young children in Ethiopia [4]. Thirty-eight percent of children age 6-23 months consumed foods rich in vitamin A, and 22% consumed iron-rich foods during the 24 h before the interview [4].

In low-income countries, diets are starchy staples containing low amounts of micronutrients, and it is challenging to obtain varieties of food groups [5]. Globally, malnutrition is the leading cause of under-five mortality accounts for 60%. In Sub-Saharan Africa, more than half of death in under-five aged children was due to malnutrition [6]. According to the 2019 Ethiopian Demographic and Health Survey (EDHS), revealed 7% of children aged 6 to 23 months meet the minimum standards concerning all three IYCF (breastfeeding status, number of food groups, and times they fed). Fourteen percent of children had an adequately diverse diet in which they had been given foods from the appropriate number of food groups, and 45% had fed the minimum meal frequency [4]. In Ethiopia, the prevalence of under nutrition has decreased considerably over the last fourteen years (2005-2019). Stunting is declined from 51 to 37%, wasting from 12 to 7%, and underweight children from 33 to 21% in the same period [3]. The age of a child between 6 to 23 months is the critical window of opportunity for their growth, and health and they need more energy and nutrient-dense foods to prevent malnutrition [1, 7]. Lack of appropriate dietary diversity (DD) in infants and young children can result in under nutrition, frequent infections, and inadequate consumption of micronutrients. The consequence could extend to poor child growth and development, weakened immune systems, and greater risk of cancer and diabetes in later life. Meanwhile, it has an enormous drain on the economy of the country [7, 8]. For instance, stunting can reduce a country’s Growth Domestic Product (GDP) by 12% [9].

Meeting the minimum standards of DD for infants and young children remains a challenge in low-income countries like Ethiopia [10]. Even though efforts have been made to improve dietary diversity feeding practice in Ethiopia, Ethiopia is still the country ranked the lowest among East African countries [11]. Previous studies conducted in Ethiopia reported the level of met MDD varies (8.5-60%) from district to district [12–16]. Furthermore, studies conducted in Ethiopia have revealed factors significantly associated with the practice of dietary diversities. These factors included mother’s/caregivers’ educational status, age, marital status, religion, occupation, family income, cost of foods, unavailability of food groups, counseling given at postnatal care, level of healthcare service, and cultural beliefs [12–16].

In Ethiopia, the quality of foods offered to children is often insufficient when compared to nutritional requirements. From the last three EDHS (2005, 2011 and 2016), dietary diversity score (DDS) among infants and young children remained low [4]. However, several studies have assessed the dietary diversity and its associated factors in different districts of Ethiopia. Since nutritional challenges are multidimensional and complex, and in Ethiopia, there is a variation in livelihood, socio-economic, culture, variety of foods served, maternal and child care practices and health services, political stability, and types of agricultural productions. Assessing dietary diversity across different regions and districts of Ethiopia is important to understand the unique factors affecting dietary diversity, to develop locally acceptable interventions to improve IYCF practices, and beneficial to decide where resource allocation is needed. In Western part of Ethiopia, there is no documented study on DD and associated factors among children aged 6-23 months in Chelia District, Ethiopia. Therefore, the purpose of the current study is to assess DD and associated factors among children aged 6 to 23 months in Chelia District, Ethiopia.

## Methods

### Study design and period

A community-based cross-sectional study design was employed to assess dietary diversity and associated factors among infants and young children aged 6 to 23 months in Chelia district, Ethiopia, from April 12th -30th, 2020. The study was conducted in the Chelia district. It is one of the districts of the West Shoa Zone and is located in the West of Ethiopia. It is about 180 from the capital city of Ethiopia, Addis Ababa. There are twenty kebeles in this district. A kebele is the smallest administrative unit in Ethiopia with approximately 1000 households [17]. According to the 2007 National Census in Ethiopia, Chelia District had 107, 429 populations. About 50.8% of them were males. The estimated under-five children were 17,618 (16.4%). The estimated number of infants and young children (6-23 months) was about 3.22%. The highest proportion of the population’s livelihood depends on agriculture. The climate of the district is categorized into highland and midland principally. The dominant food crops cultivated in the district are maize, teff, bean, pea, potato, barley, sorghum, wheat, and own livestock [18].

### Study populations

All children aged 6 to 23 months with their mothers/caregivers who are randomly selected from the selected kebeles and who met the inclusion criteria were the study population. Children aged 6 to 23 months who did not start complementary food, and were sick during the previous one week and who had a special ceremony on the day before data collection were excluded from the study.

### Sample size and sampling procedures

The proportion of children who met the recommended level of MDD, 28.5% (*p* = 0.285) [9], and confidence level = 95%, which means set at 0.05 (α = 0.05), zα/2 = 1.96 and margin of error, 5% (d = 0.05) were used to obtain 313 samples. Finally, by using correction formula including nonresponse rate (10%) and a design effect of 2, the sample size became 631.

A multi-stage sampling technique was employed to select study subjects. At the first stage, twenty kebeles were stratified into two groups: midlands and highlands, agro-ecologically. Then, at stage two, from the available five and fifteen kebeles (from the two strata), two and five kebeles were selected using simple random sampling (lottery method). Finally, the participants were selected using a systematic random sampling method based on the lists of households obtained from health posts through health extension workers. Sampling fame was constructed depending on the lists obtained for each kebeles. The lottery method was used to chosen first participant of each kebele meanwhile the subsequent respondents were determined by the sampling interval. For households having more than one child aged 6 to 23 months, a lottery method was employed to select the child. Age of child was identified based on immunization card, birth certificate or caregivers’ report. At the time of data collection, when the mother/caregiver is not available, revisit on the same day was done and if it failed, the household having the target child was considered.

The required sample of the participants from each selected kebeles was proportionally distributed. Accordingly, from Racho kebele (80), Sokondo kebele (82), Chobi kebele (102), Georges kebele (102), Dhabi kebele (88), Jarso kebele (107), and Kortu kebele (111) participants were sampled.

### Variables of the study

The dependent variable of the study was minimum dietary diversity. A minimum dietary diversity is the number of food groups consumed over a reference period; which is a proportion of infants and young children who consumed four or more food groups from the seven food groups in the previous 24 h. The seven food groups used for tabulation of this indicator were as follows: cereals, roots, and tubers; legumes and nuts; dairy products (milk, yogurt, and cheese); flesh foods (meat, fish, poultry, and liver/organ meats); eggs; vitamin A-rich fruit and vegetables and other fruits and vegetables. Children who received at least four food groups from the standard food groups recommended by the WHO without imposing a minimum intake restriction in the reference period were considered to have met MDD [1]. Children who did not receive less than four food groups from the recommended groups of food were considered to have unmet MDD [1]. Meanwhile, the independent variables of the study factors such as sex of the child, age of the child (in months), caregivers age (years), religion caregivers, marital status, occupational status, educational status, family size, family income), wealth index, physical distance, relative time for preparation, relative time for consumption, food preferences, source of food and exposure to media, IYCF information exposure during ANC or PNC follow-up, place of delivery, had information on diversified feeding, sources of information on IYCF and child health.

### Data collection tool and procedures

Data were collected using an interviewer-administered questionnaire from children of caregivers by allowing them freely to recall the type of food items they feed to their child or children within the last 24 h (24 h). The questionnaire used in this study (Additional file 1) was first prepared in the English language and translated into the Afan Oromo language. Then, translation was done in the Afan Oromo language back to English. The first part of the questionnaire contains items used to collect socioeconomic and demographic information that was adopted from the EDHS (2016) after contextual modifications were done [4]. Meanwhile, part two of a questionnaire was used to examine DD was adopted from WHO indicators for assessing IYCF practices [1]. Dietary diversity was assessed by asking the caregiver whether her child obtained food from the seven recommended seven food groups in the last 24 h. Dietary Diversity Score (DDS) ranges from zero (0) to seven (7) which is computed by summing the number of unique food groups the child received in the last 24 h.

To collect and supervise data, we recruited seven health extension workers and two supervisors. They received training on the basic principles of data collection before the actual data collection period. Pretest was conducted at Liban Jawi District, on 5% of the samples.

### Data processing and analysis

Data obtained were coded and entered into the computer using Epidata version 3.1 statistical packages and exported to SPSS version 25 for further analysis. Frequencies and cross tabulations were conducted to summarize the descriptive part of the data. Wealth index is measured by the principal component analysis and categorized as high, medium and low wealth index based on the number of assets owned by families of infant and young children. A binary logistic regression was done to identify factors associated with DD. The dependent variable (MDD) was coded as ‘1’ for those who had consumed four or more foods and ‘0’ for less than four food groups during the previous day. In the beginning, the association between each independent and dependent variable was examined by using bivariate logistic regression; then, variables that showed significant associations based on the assumptions were considered for multivariate logistic regression. Finally, in multivariate logistic regression, significant variables were identified at a *p*-value < 0.05. Data normality and multicollinearity were checked by Hosmer-Lemeshow (0.62), and variance inflation factors (VIF) indicated acceptable (less than 10).

## Results

### Socio-demographic characteristics

All 631 caregivers of children aged 6 to 23 months invited to participate in the study were successfully participated, making a response rate of 100%. More than half (58.2%) of the caregivers were female. The mean (±SD) age of children was 13.28 ± 4.18 months, and 248 (39.3%) and 267 (42.3%) were in the age group 6-11 and 12-17 months, respectively. The mean (±SD) age of the caregivers was 27 (± 6.51) years. Similarly, more than two-thirds (64.3%) of the caregivers had no formal education. More than half (58.5%) of the caregivers had a family size greater than six (Table 1).

**Table 1:** Socio-demographic characteristics of respondents and study children in Chelia District, West Shoa Zone Oromia Regional State, Ethiopia, 2020 (*n* = 631)

Variable	Categories	Number	Percentage
Child’s age (months)	6-11	248	39.3
	12-17	267	42.3
	18-23	116	18.4
Sex of the child	Male	264	41.8
	Female	367	58.2
Caregivers age (years)	15-24	248	39.3
	25-34	274	43.4
	35-44	109	17.3
Household head	Husband	436	69.1
	Wife	191	30.3
	Caregiver	4	0.6
Maternal marital status	Married	570	90.3
	Single	36	5.7
	Divorced	8	1.3
	Widowed	4	0.6
	Separate	13	2.1
Family size	1-3	70	11.1
	4-5	192	30.4
	> = 6	369	58.5
Number of under five children	1	462	73.2
	2	145	23.0
	3	24	3.8
Maternal education status	No formal education	406	64.3
	Elementary school (Grade 1-8)	154	24.4
	Secondary school (Grade 9-12)	53	8.4
	College and above	18	2.9
Maternal occupation	Housewife	202	32
	Unemployed	211	33.5
	Employer	27	4.3
	Merchant	56	8.9
	Farmer/peasant	116	18.3
	Daily laborer	19	3
Husband educational status	No formal education	285	45.2
	Primary school (Grade 1-8)	237	37.6
	Secondary school (Grade 9-12)	81	12.8
	College and above	15	2.4
Husband occupation	Unemployed	156	24.7
	Employer	49	7.8
	Merchant	75	11.9
	Farmer/peasant	278	44
	Daily laborer	73	11.6
Religion of respondent	Orthodox	135	21.4
	Protestant	482	76.4
	Others	14	2.2
Ethnicity of respondent	Oromo	597	94.6
	Amhara	18	2.9
	Others	16	2.5

^*^: Tigire, Guraghe

### Caregivers ‘healthcare service utilization and IYCF

About 20.3% of the caregivers had received information about IYCF from health extension workers. About two-thirds (60.1%) and 30.1% of caregivers had information exposure about IYCF during ANC and PNC visits, respectively. Similarly, more than three-quarters (77.2%) of mothers gave birth at health facilities. Moreover, 54.6% of caregivers reported that the infant should start complementary feeding at 4-6 months. Nearly three-fifth (59%) of the caregivers prepares food for their child in minutes not less than thirty on a day before data collection. About 56.3% of children were having food less than four times per day. About53.6% of children caregivers prefer solid food to liquid/semiliquid food for their baby. The wealth status of several caregivers (56.7%) is categorized into the lower class. Moreover, about 44% of the respondents travel more than one hour on foot to arrive at their nearby market (Table 2).

**Table 2:** Caregivers’ healthcare service utilization and IYCF in Chelia District, West Shoa Zone, Oromia Regional State, Ethiopia, 2020 (n = 631)

Variable	Categories	Frequency	Percentage
Mothers received information about IYCF during their last ANC visit	Yes	379	60.1
	No	252	39.9
Place of delivery	Hospital	148	24.9
	Health center	293	49.3
	Private clinic	22	3.7
	At home	131	22.1
Mothers received information about IYCF during their last PNC Service	Yes	190	30.1
	No	441	69.9
Had information on IYCF	Yes	347	55.0
	No	284	45.0
Sources of information about IYCF	School	43	12.4
	Friends/Relatives	52	15
	Health institutions	50	14.4
	HEW	128	36.9
	Mass media	74	21.3
Average age at which infant start complementary feeding	4-6 months	345	54.6
	After 6 months	286	45.4
Distance from market in hours	< 1 h	354	56.1
	1-2 h	210	33.3
	> 2 h	67	10.6
Average time taken to feed child yesterday in minutes	≤30 min	335	53.0
	> 30 min	296	47.0
Average time for child food preparation yesterday in minutes	≤ 30 min	259	41.0
	> 30 min	372	59.0
Household-wealth status	Low	358	56.7
	Medium	209	33.1
	High	64	10.2
Food preference	Solid	338	53.6
	Semi-solid	190	30.1
	Liquid	103	16.3
Meal frequency	≤ 3	355	56.3
	≥ 4	276	43.7

ANC: Antenatal Care; PNC: Postnatal Care; IYCF: Infant and Young Child Feeding

### Prevalence of met minimum dietary diversity

**Fig. 1:**
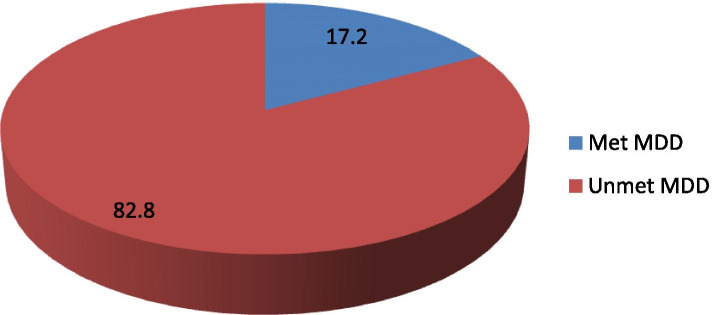
Level of dietary diversity among infants and young children aged 6 to 23 months in Chelia District, Ethiopia, 2020

**Fig. 2:**
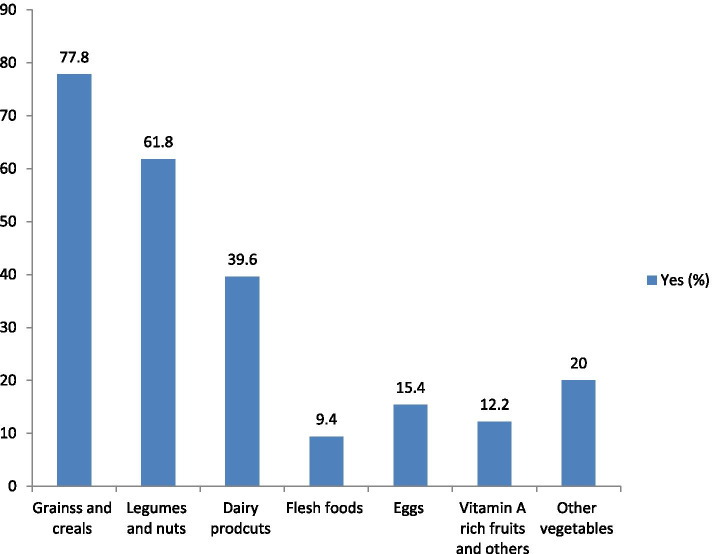
Proportion of each food group consumed in 24 hours recall by infants and young children aged 6 to 23 months in Chelia District, Ethiopia, 2020

The prevalence of infant and young children who meet the recommended minimum dietary diversity was 17.2% [95% CI (0.14-0.20)] (Fig. 1). The mean (±SD) of the minimum met the dietary diversity score (DDS) was 1.82 ± 0.015. Majorities (77.8%) of the children consume food groups composed of grains, roots, and tubers accompanied by (61.8%) legumes and nuts based on the 24 h recall (Fig. 2).

### Factors associated with MMD among children aged 6 to 23 months

In multivariate logistic regression analysis the child’s age, family size, educational status of mothers, receiving ANC follow-up, receiving counseling during PNC follow-up, household wealth status, and distance from the market remained statistically significant at *p* < 0.05.

This finding shows that the odds of meeting MDD were significantly associated and higher among children aged 18-23 months, and 12-17 months, with an adjusted odds ratio of (AOR = 3.26, 95%CI, 1.36-7.79) and (AOR = 2.85, 95%CI, 2.38-5.71), respectively, as compared to children aged 6-11 months. Mothers whose age 35-44 years old were more than three times feed their child a minimum dietary diversity than mothers aged 15-24 years old (AOR = 3.25, 95%CI1.38-7.456). Children whose household heads were housewives and caregivers who had a higher opportunity to obtain a MDD than children whose household heads are husbands, with an odds ratio of (AOR = 3.41, 95%CI, 1.567-2.379) and (AOR = 1.25, 95%CI, 3.61-5.14), respectively. Meeting minimum dietary diversity is as likely practiced among children of family size less than four compared to children of family size greater thanor equal to six (AOR=5.58, 95%CI, 1.73-17.91). Caregivers who have attended secondary school (grade 9-12) were about ten times more likely to meet minimum dietary diversity for their children than mothers who had no formal education (AOR = 9.98, 95%CI,5.66-17.10) (Table 3).

**Table 3:** Bivariate and multivariate logistic regression analysis of factors associated with minimum dietary diversity among children aged 6-23 months in Chelia District, West Shoa Zone Oromia Regional State, Ethiopia, 2020

Variables	Categories	Minimum Dietary Diversity		COR(95%CI)	AOR(95%CI)
		Met	Unmet		
Child’s age in months	06-11	32(12.9%)	216(81.1%)	1	1
	12-17	54(20.2%)	213(79.8%)	1.72(1.06-2..75)*	2.85(2.38-5.71)
	18-23	23(19.8%)	93(80.2%)	1.67(0.97-3.07)	3.26(1.36-7.79)**
Age of the mothers (years)	15-24	45(18.1%)	203(81.9%)	1	1
	25-34	38(13.9%)	236(86.1)	0.73(0.45-1.16)	0.83(0.436-1.584)
	35-44	26(23.9%)	83(76.1%)	1.42(1.82-2.43)*	3.25(1.38-7.456)**
Household head	Husband	85(19.5%)	351(80.5%)	1	1
	Housewife	24(12.6%)	167(87.4%)	0.59(0.364-0.98)*	3.41(1.567-2.379)
	Caregiver	4(40%)	6(60%)	2.75(0.76-9.97)	1.25(3.61-5.14)
Family size	1-3	6(8.6%)	64(91.4%)	0.46(0.2-0.91)*	5.58(1.73-17.91)**
	4-6	40(20.8%)	152(79.2%)	1.28(0.21-2.29)	3.89(1.18-12.78)**
	> = 6	63(17.1%)	306(82.7%)	1	1
Maternal education	No formal education	59(15.5%)	322(84.5%)	1	1
	Elementary school (Grade 1-8)	20(13.3%)	130(86.7%)	0.84(0.43-1.16)	2.45(1.09-5.49)**
	Secondary school (Grade 9-12)	25(23.5%)	52(76.5%)	2.63(1.51-4.55)*	9.98(5.66-17.10)**
	College and above	5(21.7%)	18(78.3%)	1.51(0.54-4.24)	0.64(0.36-1.45)
Mothers received information about IYCF during their last ANC visit	No	6(27.2%)	246(72.8%)	1	1
	Yes	103(2.4%)	276(97.6%)	3.3(1.21-15.48)*	1.48(2.21-11.23)*
Mothers received information about IYCF during their last PNC Service	No	49(11.1%)	392(88.9%)	1	1
	Yes	60(31.6%)	130(68.4%)	3.7(2.42-5.66)*	3.94(2.04-7.63)**
At child start complementary	4- 6 month	29(8.4%)	316(91.6%)	1	1
	> 6 month	80(28%)	206(72%)	4.23(2.67-6.70)*	3.12(1.06-2..75)
Prefer food for child	solid	14(4.1%)	324(95.9)	1	1
	semi-solid	63(33.2%)	127(66.8%)	11.48(6.21-21.23)*	8.39(1.13-6.79)**
	liquid	32(31.1%)	71(68.9)	10.13(5.29-15.56)	1.94(4.5-10.91)**
Distance from market in (hours)	< 1 h	80(19.8%)	324(80.2%)	0.58(0.31-1.07)	2.94(1.24-6.91)**
	1-2 h	12(7.1%)	158(92.9%)	0.17(0.08-0.45)*	0.29(0.132-1.62)
	> 2	17(29.8%)	40(70.2%)	1	1
Household wealth status	Low	32(9.4%)	309(90.6)	1	1
	Medium	40(19.2)	168(80.8)	2.29(1.39-3.79)*	3.34(1.09-7-54)**
	High	37(54.9%)	45(45.1%)	7.94(4.5-13.91)	4.12(1.90-8.19)**

*; represents significant variable at p-value ≤0.25 in crude odds ratio for bivariate

**: indicated significant variable at *p*-value < 0.05 in adjusted odds ratio for multivariate

COR: Crude odds ratio; AOR: Adjusted odds ratio; ANC: Antenatal care; IYCF: Infant and Young Child Feeding; PNC: Postnatal care

Likewise, receiving information about IYCF during the last ANC and PNC visit also showed a significant and positive association with the provision of minimum dietary diversity to children than those caregivers who had not received information about IYCF during the last ANC and PNC visit, with an odds ratio of (AOR = 1.48, 95%CI, 2.21-11.23) and (AOR = 3.94, 95%CI, 2.04-7.63), respectively. Similarly, caregivers who travel less than one hour to the market were about three times, children whose families had high income were four times as likely to meet dietary diversity (Table 3).

## Discussion

This cross-sectional study aimed to assess the level of met minimum dietary diversity and its associated factors in children aged 6 to 23 months in Chelia District, Ethiopia. In this study, less than one-quarter (17.2%) of children aged 6 to 23 months had offered four or more food groups. This finding is similar to reports from Ethiopia, Amara Region in Dabat District [19] and higher than the national survey (EDHS, 2016) and other study results reported in Ethiopia and India 13% [4, 17, 20, 21]. However, this finding is lower than the study results reported in Ethiopia: Wolaita zone, Bale zone, and Addis Ababa city [10, 14, 16]. The study results might vary due to differences in self-reported measurement and recalling food given in the 24 h before the survey [16]. In addition information accessible area, time of the study, and related socio-economic characteristics could also affect the estimated minimum dietary diversity score.

The study summarized that animal source foods such as eggs and flesh foods are consumed infrequently while starchy staple food groups (grains, roots and tubers) are commonly offered to children. Previously conducted studies in Ethiopia reported parallel findings [14, 16, 17]. Although eggs and other flesh products give high energy, they are less consumed in Ethiopia as they are expensive to offer children. Caregivers’ awareness about food diversity could also be another possible reason. On the other hand, starch staple foods are dominantly consumed food groups in Ethiopia due to their low cost and produced in the garden areas by several of the households.

This study showed that children aged 18-23 months offered a dietary diversity more than infants aged 6-11 months. This finding is encouraged by the study results from Haramaya town and Wolaita Zone, in Ethiopia [10, 15]. It could be since the minimum dietary diversity increases with child’s age as they are closing to switching off breastfeeding. Previous study also reported that provision of dietary diversity for children is parallel with their age [10]. Similarly, this study revealed that children of the older caregivers have a better opportunity to feed more than three food groups than children of the younger caregivers. The study findings from Kemba District also displayed similar results [22]. This possible reason might be due to caregivers’ experience in child feeding practice.

Our study also displayed that children of smaller family sizes offered a minimum dietary diversity more than larger family sizes. In Ethiopia, the majority of families have many children and lower-income. Due to low income, families might be unable to afford the required food groups. Furthermore, this study also showed that families with higher income are more dedicated to feeding a minimum dietary diversity for their children than families of low income. The studies done in the Southern region of Ethiopia (in Silte District and Wolaita Zone) displayed encouraging findings [14, 19]. This could be because of two reasons: First, families who have higher-income have no income restriction to afford the variety of food groups. Second, the majority of our participants are living in rural areas where they could have enough land to produce a variety of food groups.

Also, mothers who attended elementary/high school feed their children recommended food groups more than caregivers who have no formal education. This finding is in line with studies done in Addis Ababa city, Ethiopia [16]. It could be because educated mothers might be more likely to have more information through reading, have a hint of dietary diversity from their school courses, and have a higher ability to capture nutritional information through different mass media.

Mothers who had exposure on IYCF during their ANC offer their children dietary diversity than mothers who did not. Study findings reported in Kenya, Cambodia, and Bangladesh support this evidence [23–25]. Health information or education they obtained during their ANC visit could be the reasons. Also, this study revealed that caregivers having information about IYCF during PNC services were significantly associated with the practice of minimum dietary diversity. This evidence is similar to the previous study findings in Ghana and Tanzania [26, 27]. It could be because counseling on maternal and newborn feeding is one of the core activities at the PNC in our country, there is a positive impact on IYCF practice. Therefore, healthcare workers are encouraged to provide nutrition counseling about balanced diets and appropriate IYCF practices at the PNC clinic.

Furthermore, traveling more than an hour to reach a nearby market was also another factor influencing caregivers’ practice of feeding their baby diversity of diets. Findings from previous studies conducted in Sina District and Northwest Ethiopia [14] reported similar points. This could be mothers who live close to the market have a higher frequency of getting the diversity of food groups before the mothers who come to market after long travel and market visit. Similarly, mothers living closer to the market may have better access to health facilities and mass media.

### Study limitations

The nature of the cross-sectional study design makes it difficult to establish a cause-effect relationship. The study is not free of recall bias and social desirability bias. It may not also accurately reflect the children’s past feeding experience in the last 24-h. This study also missed valuable factors such as agro ecology of the village, access to backyard gardening, poultry production, agricultural land size, exposure to mass media messages and husband’s involvement in IYCF which thought had an impact on meeting MDD.

## Conclusions

Our study showed that consumption of minimum dietary diversity is lower than the recommended. Moreover, the child’s age, family size, maternal educational level, having ANC follow-up, receiving counseling during PNC services, distance from the market, and wealth status of families were strongly associated with minimum dietary diversity. Therefore, it is important to increase communities’ awareness on the food groups used for child growth and development by providing them appropriate information and training local representatives, and caregivers.

## Supplementary Information

**Additional file 1.**

## Data Availability

All data generated or analyzed during this study are included in this published article and its supplementary information files.

## Abbreviations

ANC: Antenatal Care
AOR: Adjusted odds ratio
COR: Crude odds ratio
DD: Dietary Diversity
EDHS: Ethiopian Demographic Health Survey
IYCF: Infant and Young Children Feeding
MDD: Minimum Dietary Diversity
PNC: Postnatal Care
SPSS: Statically Package for Social Sciences
SSA: Sub-Saharan Africa
WHO: World Health Organization
