# Supplementary material for: Dietary diversity and associated factors among children aged 6 to 23 months in Chelia District, Ethiopia
Source: BMC Pediatr. 2021 Dec 11;21:565. doi: 10.1186/s12887-021-03040-0 (PMC8665635; doi:10.1186/s12887-021-03040-0)
Supplement: Supplementary file 1 — Additional file 1. [file 12887_2021_3040_MOESM1_ESM.docx]

**ANNEX I: ENGLISH VERSION QUESTIONNAIRE**

**Part I: Socio-demographic characteristics**

| No | Questionnaires | Possible answers | Remark |
| --- | --- | --- | --- |
| 1. | What is your actual age in years? | ____________ |  |
| 2. | Age of child in a month  (Do you tell me the date that the child born?) |  |  |
| 3. | What is the sex of the child? | 1. Male  2. Female |  |
| 4. | Who is the house hold head? | 1. Husband  2. Wife  3. Caregiver  4. Other(specify) |  |
| 5. | What is your marital status? | 1.Married  2.Single  3.Divorced  4.Widowed  5.Separated |  |
| 6. | How many people are living currently in the HH? |  |  |
| 7. | How many under five children do you have currently? |  |  |
| 8. | What is your educational status mother/care taker? | 1. Unable to read and write  2. Can read and write  3. Primary school(1-8)  4. Secondary school(9-12)  5. College/University |  |
| 9 | What is the educational status of husband? | 1. Unable to read and write  2. Can read and write  3. Primary school(1-8)  4. Secondary school(9-12)  5. College/University |  |
| 10 | What is your occupation? | 1. Housewife  2. Unemployed  3.Government/NGO employee  4. Merchant  5. Farmer/peasant  6. daily laborer  7. Student  8. Other (specify).............. |  |
| 11 | What is the occupation of husband? | 1.Unemployed  2.Government/NGO employee  3.Merchant  4.Farmer/peasant  5.dailylaborer)  7.Student  8.Other (specify).................. |  |
| 12 | Average monthly house hold income | ________________ in Birr |  |
| 13 | What is your religion? | 1.Orthodox  2.Protestant  3.Wakefata  4.Muslim  5.Others(specify)________ |  |
| 14 | What is your ethnicity? | Oromo  2.Amhara  3.Tigre  4.Gurage  5.Others(Specify) |  |

**Part II: Obstetric and Child factors**

| 15 | Did you/she have ANC follow-up during pregnancy? | 1. Yes  2. No |  |
| --- | --- | --- | --- |
| 16 | Where is the place of delivery? | 1. Hospital  2. Health center  3. Private clinic  4. At home  5.Other(specify) |  |
| 17 | Did you/she have PNC follow-up and counseling? | 1. Yes  2. No |  |
| 18. | Have you ever heard any information on how to feed diversified food for your child? | 1. Yes  2. No | If no skip to Q 18 |
| 19 | If yes from where did you get the information? | l.School  2.Friends/Relatives  3.Healthinstitutions  4.Healthextensionworkers  5.Mass media  6.Others(Specify)---- |  |
| 20 | Is a mother’s or care takers sick within one week? | 1. Yes  2. No | If no skip to Q 20 |
| 21 | If yes what type illness/sick? | 1.cough  2.diarrhea  3.fever  4.Others (specify)___ |  |
| 22 | Do you drinking alcohol currently? | 1. Yes  2. No |  |
| 23 | Do you chat chewing? | 1. Yes  2. No |  |
| 24 | Do you smoking? | 1. Yes  2. No |  |

| 25 | Is the child breast feeding? | 1. Yes  2. No |  |
| --- | --- | --- | --- |
| 26 | At what age the child started complementary food? | 1. 4-6 month  2. >6 month |  |
| 27 | How many children under five in house? | 1.One  2.two  3.three  4.four |  |
| 28 | Is a child sick in within two weeks? | 1.yes  2.No | If no skip to Q 28 |
| 29 | If yes what type illness/diseased? | 1.Diarrhoea  2. Cough  3.Fever  4. Others |  |
| 30 | Is a child is vaccinated? | 1.yes  2.No | If no skip to Q 30 |
| 31 | If yes for vaccination? | 1.Up to date  2.fully vaccinated  3.Defualted  4.Others (specify) |  |

**Part III: Factors dietary diversify foods in House**

32. How many of the following assets are owned by your house hold (HH)? (Only functioning assets)

| No | Asset in house function |  | No | Asset in house function |  |
| --- | --- | --- | --- | --- | --- |
| 1 | Shelf/cabinet | 1.Yes  2.No | 10 | Grains \roots  (in quintal) | 1.Yes  2.No |
| 2 | Sofa Set | 1.Yes  2.No | 11 | Horse/donkey | 1.Yes  2.No |
| 3 | Radio | 1.Yes  2.No | 12 | Cart | 1.Yes  2.No |
| 4 | Television | 1.Yes  2.No | 13 | Cows | 1.Yes  2.No |
| 5 | Satellite dish | 1.Yes  2.No | 14 | Motorcycle/bicycle | 1.Yes  2.No |
| 6 | Goat/sheep | 1.Yes  2.No | 15 | Car | 1.Yes  2.No |
| 7 | Large electric stove/mitad | 1.Yes  2.No | 16 | Cell phone | 1.Yes  2.No |
| 8 | Small gas stove | 1.Yes  2.No | 17 | Legumes and nuts(in quintal) | 1.Yes  2.No |
| 9 | Refrigerator/freezer | 1.Yes  2.No | 18 | watch/clock | 1.Yes  2.No |

31. What are your main sources of food to feed the child?

| No | Food group | Source of food | | | | |
| --- | --- | --- | --- | --- | --- | --- |
|  |  | Own production | Casual labor/work | By Aid | From market | Other  (specify) |
| 1 | grains, roots and tubers |  |  |  |  |  |
| 2 | Legumes and nuts |  |  |  |  |  |
| 3 | dairy products (milk,  yogurt,cheese) |  |  |  |  |  |
| 4 | flesh foods(meat, fish,  poultry and liver/organ meats) |  |  |  |  |  |
| 5 | Eggs |  |  |  |  |  |
| 6 | vitamin-A rich fruits and  vegetables |  |  |  |  |  |
| 7 | other fruits and  vegetables |  |  |  |  |  |

33. If it is from market/shop how many hours does it take to purchase the food item? (hr)___

34. How many minutes did (relative time) it takes for preparation of food for child yesterday on average?

35. How many minutes did (relative time) it take for consumption of food after preparation yesterday on average?

35. Which food item do group prefer for the child feeding?

1. Solid

2. Semi-solid

3. Liquid

**Part. IV Dietary Diversity Questionnaire**

| S.No. | Food group | Examples | Yes=1mention if“Yes”  No=0 |
| --- | --- | --- | --- |
| 1 | GRAINS,ROOTSANDTUBERS(CEREALS) | corn/maize,teff,beans,wheat,sorghum,milletor any other grains or foods made from these (e.g.bread,injera,porridge or other grain products)  white potatoes (Kocho),white yams,white cassava,or other foods made from roots |  |
| 2 | LEGUMESANDNUTS | beans,peas,lentils,nuts,seeds or foods made from these |  |
| 3 | DAIRY PRODUCTS | milk,cheese,yogurt or other milk products  fats or butter added to food or used for cooking |  |
| 4 | FLESH FOODS | liver,kidney,heart or other organ meats or  blood-basedfoods beef,pork,goat,chicken,duck,or other birds fresh or dried fish |  |
| `5 | EGGS | chicken,duck,or any other egg |  |
| 6 | VITAMIN-A RICHFRUITSAND  VEGETABLES | Ripe mangoes,cantaloupe,ripe papaya,  Dried peaches other locally available vitamin A rich fruits pumpkin,carrots,squash,orsweet potatoes that are orange insideothers |  |
| 7 | OTHERFRUITS ANDVEGETABLES | Other vegetables (e.g.tomato,onion),including wild vegetables  Dark green/leafy vegetables,including wildoneslocally available vitamin ,etc. |  |

**Part V: Households food security Questionnaire**

| S/no | Questionnaire HHFS | Options/Response |
| --- | --- | --- |
| 1 | In the past four weeks, did you worry that your household would not have enough food? | 0 = No (skip to Q2)  1=Yes |
| 1a | How often did this happen? | 1 = Rarely (once or twice in the last 4 weeks )  2 = Sometimes (3-10 times)  3 = Often (more than ten times) |
| 2 | In the past four weeks, were you or any household member not able to eat the kinds of foods you preferred because of a lack of resources? | 0 = No (skip to Q3)  1=Yes |
| 2a | How often did this happen? | 1 = Rarely (once or twice in the last 4 weeks )  2 = Sometimes (3-10 times)  3 = Often (more than ten times) |
| 3 | In the past four weeks, did you or any household member have to eat a limited variety of foods due to a lack of resources? | 0 = No (skip to Q4)  1=Yes |
| 3a | How often did this happen? | 1 = Rarely (once or twice in the last 4 weeks )  2 = Sometimes (3-10 times)  3 = Often (more than ten times) |
| 4 | In the past four weeks, did you or any household member have to eat some foods that you really did not want to eat because of a lack of resources to obtain other types of food? | 0 = No (skip to Q5)  1=Yes |
| 4a | How often did this happen? | 1 = Rarely (once or twice in the last 4 weeks )  2 = Sometimes (3-10 times)  3 = Often (more than ten times) |
| 5 | In the past four weeks, did you or any household member have to eat a smaller meal than you felt you needed because there was not enough food? | 0 = No (skip to Q6)  1=Yes |
| 5a | How often did this happen? | 1 = Rarely (once or twice in the last 4  weeks )  2 = Sometimes (3-10 times)  3 = Often (more than ten times) |
| 6 | In the past four weeks, did you or any household member have to eat fewer meals in a day because there was not enough food? | 0 = No (skip to Q7)  1=Yes |
| 6a | How often did this happen? | 1 = Rarely (once or twice in the last 4  weeks )  2 = Sometimes (3-10 times)  3 = Often (more than ten times) |
| 7 | In the past four weeks, was there ever no food to eat of any kind in your household because of lack of resources to get food? | 0 = No (skip to Q8)  1=Yes |
| 7a | How often did this happen? | 1 = Rarely (once or twice in the last 4 weeks )  2 = Sometimes (3-10 times)  3 = Often (more than ten times) |
| 8 | In the past four weeks, did you or any household member go to sleep at night hungry because there was not enough food? | 0 = No (skip to Q9)  1=Yes |
| 8a | How often did this happen? | 1 = Rarely (once or twice in the last 4 weeks )  2 = Sometimes (3-10 times)  3 = Often (more than ten times) |
| 9 | In the past four weeks, did you or any household member go a whole day and night without eating anything because there was not enough food? | 0 = No (skip )  1=Yes |
| 9a | How often did this happen? | 1 = Rarely (once or twice in the last 4 weeks )  2 = Sometimes (3-10 times)  3 = Often (more than ten times) |

Thank you!
